# Spatial modulation with design of expanded antenna index vectors in MIMO communication networks

**DOI:** 10.1038/s41598-026-46570-2

**Published:** 2026-04-10

**Authors:** Shuqing Lin, Yanfei Zhu, Zhi Yang, Fuchun Huang

**Affiliations:** 1 Guangzhou College of Commerce, Guangzhou, 511363 China; 2 Hubei Normal University, Huangshi, 435002 China; 3 Sun Yat-sen University, Guangzhou, 510275 China; 4 Tencent Technology Company Limited, Shenzhen, 518000 China

**Keywords:** Expanded antenna index vectors, Three dimension (3D), AI set, Squared minimum euclidean distance (MED), Electrical and electronic engineering, Energy science and technology, Engineering

## Abstract

Spatial index modulation, which utilizes the antenna spatial domain to exploit the additional information, is a promising technique for improving the spectral efficiency in next wireless communication network. In this paper, to exploit more additional information from the antenna spatial domain, with the dimensions of the three dimension (3D) signal constellation, Space Modulation with design of Expanded antenna Index Vectors (EIV) by modulating Two types of 3D signal Constellations (SM-EIV-T3DC) is developed. In the proposed SM-EIV-T3DC, with the aid of two types of 3D signal constellations, an extended antenna index (AI) vector set $\Gamma$ is first designed, in which one part of vectors is used to modulate the conventional 3D signal constellation points (CPs) and another part of vectors is used to modulate the secondary 3D signal CPs. Then, on the basis of modulating three components of one 3D signal CP by the designed set $\Gamma$, four AI vector sets: $\{\Upsilon _1, \Upsilon _2,\Upsilon _3,\Upsilon _4\}$ are designed to expand the number of AI vectors, whose vectors contain one or two non-zero elements equaling to ”j”. All vectors from two sets $\{\Upsilon _1, \Upsilon _3\}$ are with two non-zero elements, while all vectors from two sets $\{\Upsilon _2, \Upsilon _4\}$ only contain one non-zero element. Furthermore, the specified vector from the set $\Gamma$ with one part of AI bits and the specified vector from the set $\Upsilon _\ell , \ell \in \{1,2,3,4\}$ with the other one part of AI bits are constructed into one space vector to modulate one 3D signal CP symbol, resulting in one transmitted space vector (TSV). Furthermore, to increase the squared minimum Euclidean distance between the TSVs, the modified secondary 3D signal constellation is designed. Finally, the bit error probability is analyzed and experimental verifications are provided to prove that the proposed SM-EIV-T3DC outperforms the existing schemes such as signed quadrature spatial modulation (SQSM), spatial modulation with spatial constellation (SM-SC), quadrature index modulation with three dimension constellation (QIM-TDC) in terms of bit error rate (BER) performance.

## Introduction

Index modulation (IM), which is viewed as one of the key techniques in multi-input multi-output (MIMO) systems, has a greatly potential to exploit the additional information for improving the spectral efficiency (SE) in next-generation wireless communications. In 2008, on the basis of the MIMO spatial domain, the spatial modulation (SM)^1^ scheme is proposed to carry the additional information by exploiting the spatial domain of transmit antennas (TAs), i.e., developing the third dimension for increasing the SE. Furthermore, in order to improve the bit error rate (BER) performance, based on the lattice method, spatial lattice modulation^2^ is designed to enhance the squared minimum Euclidean distance (MED) between the transmit vectors. However, the number of additional information in the SM system is base-two logarithm of the number of TAs. Based on this, the SM is unrealistic for the massive MIMO systems. From this, the generalized SM (GSM)^3–5^, in which several TAs are activated simultaneously to convey different or same signal constellation point(s) (CP) from the QAM/PSK constellation, is proposed to not only relax the number of TAs but also improve the SE. In Ref^3^., one signal CP is transmitted by multiple active TAs for the diversity gain, while multiple different signal CPs are transmitted by multiple active TAs for the multiplex gain^4,5^. Furthermore, utilizing the interpolating method on the design of multiple signal constellations, the enhanced spatial modulation (ESM) scheme^6,7^ is proposed to improve the squared MED for enhancing the BER performance. In the ESM scheme, the antenna index (AI) vector with one non-zero element is used for the modulating of conventional QAM CP, and the AI vector with two non-zero elements is used for the modulating of two secondary QAM CPs. Although the ESM achieves the better BER performance than the quadrature spatial modulation (QSM)^8^, the developing of the additional information is limited to only one spatial domain. In Ref^8^., combining with the quadrature and in-phase dimensions of signal CP, the QSM develops the quadrature and in-phase spatial domains for carrying twofold base-two logarithm of the number of TAs.

To further enhance the SE and reliability of MIMO systems, a lot of works have been done by exploiting the in-phase and quadrature spatial dimensions. On the one hand, the variants of QSM have been developed into multiple schemes such as improved QSM (IQSM)^9–11^ and complex QSM (CQSM)^12,13^. In Ref^9–11^., the improved QSM (IQSM) activates two transmit antennas in quadrature/in-phase dimension to convey the real/imaginary parts of two signal symbols, respectively. The CQSM and double SM schemes introduce angle rotation to transmit two different QAM/PSK symbols via one active antenna in each dimension. To exploit more index domains, the permutation index QSM (PI-QSM)^14^ was developed by incorporating the permutation index domain. Furthermore, to fully utilize the available transmit antennas, parallel transmission schemes such as parallel QSM (PQSM)^15^ and parallel CQSM (PCQSM)^16^ were proposed via antenna grouping. Beyond index design, signal constellation optimization has also been explored. For instance, irregular Euclidean distance QAM/PSK (IEDQAM/PSK)^17^ was designed for quadrature index modulation schemes to improve the BER performance. The achievable rate analysis of generalized quadrature spatial modulation and the development of low-complexity detectors have provided valuable insights into the performance-complexity trade-offs in such systems^18^. On the other hand, the combination of active antenna indices and advanced signal constellation design has proven to be a fruitful research direction. The quadrature index modulation with three-dimensional constellation (QIM-TDC)^19^ leverages the in-phase and quadrature dimensions to design a 3D constellation, thereby enhancing the squared MED between transmit vectors. By utilizing the AI combinations of arbitrary number of active TAs, the double GSM^20^ extends the number of antenna index vectors in the in-phase and quadrature dimensions to modulate the same signal constellation point, improving both SE and diversity gain. Recently, the multiple constellations and variable active antennas selection for signal spaces design (SSD-MCVA)^21^ was proposed to exploit the spatial domain and expand the signal spaces for carrying more additional information bits, albeit at the cost of increased implementation complexity. However, the complexity of implementation may be high. It is worth noting that the optimization of the physical propagation environment itself, such as through movable antennas^22–24^ or reconfigurable intelligent surfaces (RIS)^25–27^, has emerged as a powerful complementary approach. For example, fluid antenna systems^22^ can minimize the total transmit power or maximize the secrecy rate^23^, while RIS-assisted movable antenna systems^24^ can significantly improve the communication quality. K-means based constellation optimization for index-modulated RIS further demonstrates the potential of joint optimization in the index and signal domains^26^. However, these approaches primarily focus on reshaping the channel and do not directly develop the spatial index domains of the transmit antennas for improving SE.

In addition to the above, extending the index domains is another effective strategy for enhancing SE. The signed QSM (SQSM)^28^evolves from QSM by extending to four spatial domains, thereby offering a fourfold logarithm of the number of TAs. However, this expansion comes at the cost of a reduced number of signal constellation points. In Ref^29^., to retain the advantage of the un-normalized squared MED (i.e., $d^2_\mathrm{{dim}}=4$) between the transmit vectors in the SM and GSM systems, the generalized SM with multi-index modulation (GSM-MIM) was designed by employing two types of signal constellations, such as secondary PAM (e.g., 8-SPAM: $\left\{ { \pm 2 \pm 2j, \pm 2 \pm 4j} \right\}$) and conventional QAM. The spatial modulation with spatial constellation (SM-SC)^30^ exploits the symbol group index domain with the signs $''1''$ and $''j''$ to enhance the transmission of additional information. More recently, the extended space index modulation (ESIM)^31^ was designed to increase the number of antenna index vectors by employing two types of signal constellations, such as QAM and secondary QAM, while retaining the merit of QSM. Furthermore, to jointly exploit multiple index domains, the spatial modulation with joint permutation, group, and antenna indexes (JPGA-ISM)^32^ was proposed.

### Motivation and contribution

In this paper, inspired by the design ideas of these schemes such as the ESM^6^, the QIM-TDC^19^ and the ESIM^31^, a new design of $\textbf{S}$pace $\textbf{M}$odulation with design of $\textbf{E}$xpanded antenna $\textbf{I}$ndex $\textbf{V}$ectors (EIV) by modulating $\textbf{T}$wo types of $\textbf{3D}$ signal $\textbf{C}$onstellations ($\mathbf {SM\mathrm{-}EIV\mathrm{-}T3DC}$), which further develops the quadrature and in-phase spatial domains for exploiting the additional information, is proposed to design the spatial vectors with three non-zero elements for conveying one of two types of 3D signal CPs. The main contributions of the proposed SM-EIV-T3DC are summarized as below:

1. The design framework of the SM-EIV-T3DC system is developed, which expands the signal spaces by combing the design of AI vectors with two types of 3D signal constellations. In order to expand the spatial index domain, on the basis of three components of one 3D signal CP, the AI vectors formed by two non-zero elements in the in-phase or quadrature spatial dimensions of transmit antennas are designed to modulate two components of one 3D signal CP from two types of 3D signal constellations. Similarly, the AI vectors formed by one non-zero element in the in-phase or quadrature spatial dimensions are designed to modulate the remaining one component. Thus, compared with the previous works, the AI vectors are further developed to enhance the ability to carry additional information.
2. Firstly, an AI vector set $\Gamma =\{\Gamma _1,~\Gamma _2,~\Gamma _3,~\Gamma _4\}$, the number of AI vectors in which satisfy the power of two, is designed, where two subsets of $\{\Gamma _1,~\Gamma _3\}$ are constructed by all vectors with one non-zero elements equaling to ”1”, and the other two subsets of $\{\Gamma _2,~\Gamma _4\}$ are with the vectors having two non-zero elements equaling to ”1”. Furthermore, to convey three components of one 3D symbol, four AI vector sets: $\{\Upsilon _1, \Upsilon _2,\Upsilon _3,\Upsilon _4\}$ are designed, where non-zero elements of all AI vectors equaling to ”j”. Specifically, on the basis of modulating one/two components of one conventional 3D (C3D) constellation through the specified vector from the subset of $\Gamma _1$/$\Gamma _2$, the AI vector set $\Upsilon _1$/$\Upsilon _2$ are designed to modulate the other two/one component from the C3D constellation. Then, in order to develop the number of AI vectors, the specified vector from the subset of $\Gamma _3$/$\Gamma _4$ is used to modulate one/two components from the secondary 3D (S3D) constellation, thus the AI vector set $\Upsilon _3$/$\Upsilon _4$ are designed to modulate the other two or one component from the S3D constellation. Consequently, three components of one 3D symbol are modulated on corresponding active TAs, forming multiple types of the transmitted space vector (TSV) to be transmitted.
3. In addition, on basis of analyzing the squared MED in the MIMO-IM systems, the modified S3D constellations are designed to maximum the squared MED by combining with the C3D constellations. Furthermore, the formula of the average bit error probability (BEP) is provided.
4. Simulation results with maximum likelihood (ML) detector show that, (1). The theoretical results are in agreement wit the simulation results under the same configurations in the high signal noise ratio (SNR) region. (2). Experimental verifications such as better BER performance and robustness are provided to prove that the SM-EIV-T3DC has the advantage in comparisons with other existing schemes such as the SQSM, QIM-TDC and SM-SC schemes.

The organization of this paper proceeds as below. Section System model depicts the system model of the SM-EIV-T3DC. In Sect. Methods design, the design of the AI vector Sets are provided. Section Performance analysis provides the analysis of system. Simulation results are discussed in Sect. Simulation and numerical results and our conclusions are given in Sect. Conclusions.

## System model

In this paper, the objective of our design is to further exploit the additional information by jointly designing of the antenna index combinations of the three active transmit antennas in the quadrature and in-phase dimensions with two types of 3D constellations, and to maximize the squared MED between the transmitted spatial vectors (TSVs).

**Fig. 1:**
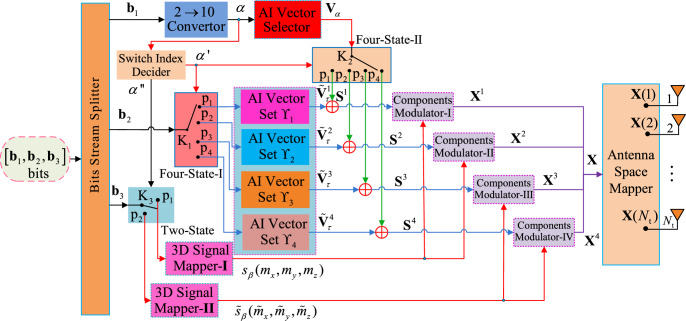
Transmitter of the proposed SM-EIV-T3DC.

The transmitter of this proposed SM-EIV-T3DC is presented in Fig. 1.

### Transmitter

Through the Bits Stream Splitter, as is described in Fig. 1, the input block of $[\mathbf{b_1},~\mathbf{b_2},~\mathbf{b_3}]$ information bits is split into three subblocks: $\textbf{b}_1$, $\textbf{b}_2$, $\textbf{b}_3$. For the subblock of $\textbf{b}_1$, with $\log _2 \left| \Gamma \right|$ bits, it is for the selection of the AI vectors from the AI vector set $\Gamma$ that will be introduced in the following, $\left| \Gamma \right|$ is the number of AI vector in the set $\Gamma$. For the subblock of $\textbf{b}_2$, with $\log _2 \left| \Upsilon _\kappa \right|$ ($\kappa \in {1, 2, 3, 4}$) bits, it is for the selecting one out of the AI vectors in selected AI vector set $\Upsilon _1$, or $\Upsilon _2$, or $\Upsilon _3$, or $\Upsilon _4$, each of the four AI vector sets has the same number of the AI vectors, where $\left| \Upsilon _\kappa \right|$ is the number of the vectors in the set $\Upsilon _\kappa$. For the subblock of $\textbf{b}_3$, with $\log _2 M$ bits, it is for the mapping of one signal constellation point (CP) $s(m_x, m_y, m_z)$ from the selected 3D signal CPs set $\Omega _\textrm{I}$ or $\Omega _\textrm{II}$ that will be provided in next subsection.

Specifically, the modulating process of three subblocks: $\textbf{b}_1$, $\textbf{b}_2$, $\textbf{b}_3$ are provided in detail in the following.

With the beginning, the subblock of $\textbf{b}_1$, containing $\log _2 \left| \Gamma \right|$ bits, is fed to the $2 \rightarrow 10$ Convertor and then into a decimal number $\alpha$. Then, the decimal number $\alpha$ is fed not only into the AI Vector Selector but also into the Switch Index Decider. In the AI Vector Selector, the decimal number $\alpha$ is used to select an AI vector $\textbf{V}_\alpha$ from an AI vector set $\Gamma = \left\{ {\Gamma _1 ,\Gamma _2 ,\Gamma _3 ,\Gamma _4 } \right\}$, note that the number of all vectors in the AI vector set $\Gamma$ satisfies the power of two and is denoted by $\left| \Gamma \right|$, its detailed designs will be provided in next section. In addition, the decimal number $\alpha$ plays an important role in the controlling three Switches of $\textrm{K}_1,~\textrm{K}_2,~\textrm{K}_3$. According to the decision of the size of $\alpha$, the Switch Index Decider converts the decimal number $\alpha$ into two decimal number values, i.e., $\alpha '$ and $\alpha ''$, as is shown in Fig. 1, $\alpha '$ for controlling both of the Switch $\textrm{K}_1$ and $\textrm{K}_2$ and $\alpha ''$ for controlling the Switch $\textrm{K}_3$. Specifically, according to the size of the decimal number $\alpha$, the Switch Index Decider has two cases of working mechanisms, as follows:

$\textbf{Case} 1:$ For the output value of the parameter $\alpha ''$,

1. If $\alpha \le \left| {\Gamma _1 } \right| + \left| {\Gamma _2 } \right|$, the output parameter: $\alpha ''=1$, where $\left| {\Gamma _1 } \right|$ and $\left| {\Gamma _2 } \right|$ are the number of the AI vectors in the sets of both $\Gamma _1$ and $\Gamma _2$, respectively. In this case, the Switch $\mathrm{K_3}$ links the key p_1_ in the module of Two-State. Such that the subblock of $\textbf{b}_3$ bits is fed into the 3D Signal Mapper-$\textbf{I}$. After that, the $\textbf{b}_3$ bits are mapped into one *M*-ary 3D signal CP $s_\beta (m_x, m_y, m_z)$ from the 3D CPs set $\Omega _\textrm{I}$, where $\beta$ is the decimal number that is converted from the $\textbf{b}_3$ bits.
2. If $\alpha> \left| {\Gamma _1 } \right| + \left| {\Gamma _2 } \right|$, the output value $\alpha ''=2$. In this case, the Switch $\mathrm{K_3}$ controlled by the output $\alpha ''$ links the port p_2_ in the module of Two-State and then feeds the subblock of $\textbf{b}_3$ bits into the 3D Signal Mapper-$\textbf{II}$. Then, the subblock of $\textbf{b}_3$ bits is mapped into the 3D signal CP ${\tilde{s}}_\beta ({\tilde{m}}_x, {\tilde{m}}_y, {\tilde{m}}_z)$ from the signal CPs set $\Omega _\textrm{II}$.

$\textbf{Case} 2:$ For the second output of parameter $\alpha '$ in the Switch Index Desider, it is important to note that the $\alpha '$ is used not only to control the Switch $\mathrm{K_1}$ but also the Switch $\mathrm{K_2}$. Moreover, with the control of the parameter $\alpha '$, both of $\mathrm{K_1}$ and $\mathrm{K_2}$ link the corresponding port ${\mathrm{p}}_{{\alpha ^{\prime } }}$ with the same index $\alpha '$ in the Four-State-$\textbf{I}$, -$\textbf{II}$, respectively. Specifically, according to the size of the parameter $\alpha$, the decision rule of the Switch Index Decider for the output value of the value $\alpha '$ is as follows:

1. If $1 \le \alpha \le \left| {\Gamma _1 } \right|$, the output value $\alpha '=1$. With the control of the parameter $\alpha '$, the two Switches: $\mathrm{K_1}$ and $\mathrm{K_2}$ controlled by the parameter $\alpha '$ simultaneously link the port $\textrm{p}_1$ in the Four-State-$\textbf{I}$, -$\textbf{II}$, respectively.
2. Similarly, if $\left| {\Gamma _1 } \right| +1 \le \left| {\Gamma _1 } \right| +\left| {\Gamma _2 } \right|$, the output value $\alpha '=2$. In this case, two Switches (i.e., $\mathrm{K_1}$ and $\mathrm{K_2}$) simultaneously link the port $\textrm{p}_2$ in the Four-State-$\textbf{I}$, -$\textbf{II}$, respectively.
3. If $\left| {\Gamma _1 } \right| +\left| {\Gamma _2 } \right| +1 \le \left| {\Gamma _1 } \right| +\left| {\Gamma _2 } \right| +\left| {\Gamma _3 } \right|$, the output value $\alpha '=3$. In this case, with the control of the parameter $\alpha '$, two ports (i.e., $\textrm{p}_3$) in the Four-State-$\textbf{I}$, -$\textbf{II}$ are linked by the two Switches: $\mathrm{K_1}$ and $\mathrm{K_2}$, respectively.
4. If $\left| {\Gamma _1 } \right| +\left| {\Gamma _2 } \right| +\left| {\Gamma _3 } \right| +1 \le \alpha \le \left| {\Gamma } \right|$, the output value $\alpha '=4$. In this case, two ports ( i.e., $\textrm{p}_4$) in the the Four-State-$\textbf{I}$, -$\textbf{II}$ are selected to be linked by the two Switches $\mathrm{K_1}$ and $\mathrm{K_2}$, respectively.

More specifically, with the control of the parameter $\alpha '$, the subblock of $\textbf{b}_2$ bits are fed by the Switch K_1_ in the Four-State-$\textbf{I}$ into the ${\alpha '}$-th module equipped with in the AI vector set $\Upsilon _{\alpha '}, \alpha ' \in \{1,2,3,4\}$ over the port ${\mathrm{p}}_{{\alpha ^{\prime } }}$, where the design method of $\{\Upsilon _1, \Upsilon _2,\Upsilon _3,\Upsilon _4\}$ will be introduced in Sect. 3.2. Then, the AI vector ${\tilde{\textbf{V}}}_\tau ^{\alpha '}$ from the set $\Upsilon _{\alpha '}=\left\{ {{ \tilde{\textbf{V}}}_1^{\alpha '},{ \tilde{\textbf{V}}}_2^{\alpha '}, \cdots ,{\tilde{\textbf{V}}}_{N_\textrm{t}} ^{\alpha '} } \right\}$ is selected, where $\tau$ is the decimal number converted from the $\textbf{b}_2$ bits, $\gamma$ satisfies the power of two. Similarly, with the control of the $\alpha '$, the AI vector $\textbf{V}_\alpha$ passes through the Switch K_2_ linking the key ${\mathrm{p}}_{{\alpha ^{\prime } }}$ in the Four-State-$\textbf{II}$. On the basis of the above design, utilizing the $\alpha '$-th Adder, the vector $\textbf{V}_\alpha$ is added to the specified AI vector ${\tilde{\textbf{V}}}_\tau ^{\alpha '}$, resulting in the spatial vector $\textbf{S}^{\alpha '}=\textbf{V}_\alpha +{\tilde{\textbf{V}}}_\tau ^{\alpha '}$ with three non-zero components to be fed into the $\alpha '$-th Components Modulator-$\alpha '$. Furthermore, the Components Modulator-$\alpha '$ utilizes the resulted spatial vector $\textbf{S}^{\alpha '}$ with three non-zero elements to modulate three components (e.g., $m_x,m_y,m_z$) of the mapped 3D signal CP $P(m_x,m_y,m_z), P \in \{s, \tilde{s}\}$, resulting in one TSV $\textbf{X}$, which can be expressed as

1 $$\begin{aligned} \begin{array}{l} \textbf{X}\begin{array}{*{20}c} = \\ \end{array} \textbf{X}^\Re + \textbf{X}^\Im \\ \begin{array}{*{20}c} {} \\ \end{array} = \left\{ \begin{array}{l} \textrm{Mod}(\textbf{V}_\alpha ,m_x ) + \textrm{Mod}({\tilde{\textbf{V}}}_\tau ^1 ,\{ m_y ,m_z \} ),\begin{array}{*{20}c} {} \\ \end{array}1 \le \alpha \le \left| {\Gamma _1 } \right| \\ \textrm{Mod}(\textbf{V}_\alpha ,\{ m_x ,m_y \} ) + \textrm{Mod}({\tilde{\textbf{V}}}_\tau ^2 ,m_z ),\begin{array}{*{20}c} {} \\ \end{array}\left| {\Gamma _1 } \right| + 1 \le \alpha \le \left| {\Gamma _1 } \right| + \left| {\Gamma _2 } \right| \\ \textrm{Mod}(\textbf{V}_\alpha ,\tilde{m}_x ) + \textrm{Mod}({\tilde{\textbf{V}}}_\tau ^3 ,\{ \tilde{m}_y ,\tilde{m}_z \} ),\begin{array}{*{20}c} {} \\ \end{array}\left| {\Gamma _1 } \right| + \left| {\Gamma _2 } \right| + 1 \le \alpha \le \left| {\Gamma _1 } \right| + \left| {\Gamma _2 } \right| + \left| {\Gamma _3 } \right| \\ \textrm{Mod}(\textbf{V}_\alpha ,\{\tilde{m}_x ,\tilde{m}_y \} ) + \textrm{Mod}({\tilde{\textbf{V}}}_\tau ^4 ,\tilde{m}_z ),\begin{array}{*{20}c} {} \\ \end{array}\left| {\Gamma _1 } \right| + \left| {\Gamma _2 } \right| + \left| {\Gamma _3 } \right| + 1 \le \alpha \le \left| {\Gamma } \right| \\ \end{array} \right. \\ \begin{array}{*{20}c} {} \\ \end{array} = \left\{ \begin{array}{l} \textbf{e}_\alpha \cdot m_x + (\textbf{e}_{\tau _1 } \cdot m_y \mathrm{+ } \textbf{e}_{\tau _2 } \cdot m_z ),\begin{array}{*{20}c} {} & {} \\ \end{array}1 \le \alpha \le \left| {\Gamma _1 } \right| \\ (\textbf{e}_{\alpha _1 } \cdot m_x + \textbf{e}_{\alpha _2 } \cdot m_y \mathrm{) + } \textbf{e}_\tau \cdot m_z ,\begin{array}{*{20}c} {} & {} \\ \end{array}\left| {\Gamma _1 } \right| + 1 \le \alpha \le \left| {\Gamma _1 } \right| + \left| {\Gamma _2 } \right| \\ \textbf{e}_\alpha \cdot \tilde{m}_x + \textbf{e}_{\tau _1 } \cdot \tilde{m}_y \mathrm{+ } \textbf{e}_{\tau _2 } \cdot \tilde{m}_z ),\begin{array}{*{20}c} {} & {} \\ \end{array}\left| {\Gamma _1 } \right| + \left| {\Gamma _2 } \right| + 1 \le \alpha \le \left| {\Gamma _1 } \right| + \left| {\Gamma _2 } \right| + \left| {\Gamma _3 } \right| \\ (\textbf{e}_{\alpha _1 } \cdot \tilde{m}_x + \textbf{e}_{\alpha _2 } \cdot \tilde{m}_y \mathrm{) + } \textbf{e}_\tau \cdot \tilde{m}_z ,\begin{array}{*{20}c} {} & {} \\ \end{array}\left| {\Gamma _1 } \right| + \left| {\Gamma _2 } \right| + \left| {\Gamma _3 } \right| + 1 \le \alpha \le \left| {\Gamma } \right| \\ \end{array} \right. , \\ \end{array} \end{aligned}$$

where Mod($\cdot$) denotes the modulating operation. $\tau _1$ and $\tau _2$ are the row number of the non-zero elements in the selected AI vector $\tilde{\textbf{V}}^1_\tau$ or $\tilde{\textbf{V}}^3_\tau$, $\alpha _1$ and $\alpha _2$ are the row number of the non-zero elements in the selected AI vector $\textbf{V}_\alpha$.

Finally, through the Antenna Space Mapper, $$N_\textrm{t}$$ components of the resulted TSV $$\textbf{X}$$ are mapped on the corresponding $$N_\textrm{t}$$ TAs to be transmitted. For the sake of clarity and intuitiveness, based on the above-mentioned design of the proposed SM-EIV-T3DC, some examples of forming the TSV $$\textbf{X}$$ are given in Table 1, where assumed that the mapped 3D constellation symbol $$s(m_x,m_y,m_z)$$ or $$\tilde{s}(\tilde{m}_x,\tilde{m}_y,\tilde{m}_z)$$, $$N_t=4$$. Thus, the AI vector set $$\Gamma$$ is given as Eq. (6). Also, according to the design principle of subsection 3.2, four AI vector sets: $$\{\Upsilon _1$$, $$\Upsilon _2$$, $$\Upsilon _3$$, $$\Upsilon _4\}$$ can be respectively obtained as $$\Upsilon _{1} = \left\{ {\left[ {\begin{array}{*{20}c} j \\ 0 \\ j \\ 0 \\ \end{array} } \right],\left[ {\begin{array}{*{20}c} j \\ 0 \\ 0 \\ j \\ \end{array} } \right],\left[ {\begin{array}{*{20}c} 0 \\ j \\ j \\ 0 \\ \end{array} } \right],\left[ {\begin{array}{*{20}c} 0 \\ j \\ 0 \\ j \\ \end{array} } \right]} \right\},$$$$\Upsilon _{2} = \left\{ {\left[ {\begin{array}{*{20}c} j \\ 0 \\ 0 \\ 0 \\ \end{array} } \right],\left[ {\begin{array}{*{20}c} 0 \\ j \\ 0 \\ 0 \\ \end{array} } \right],\left[ {\begin{array}{*{20}c} 0 \\ 0 \\ j \\ 0 \\ \end{array} } \right],\left[ {\begin{array}{*{20}c} 0 \\ 0 \\ 0 \\ j \\ \end{array} } \right]} \right\},$$$$\Upsilon _{3} = \left\{ {\left[ {\begin{array}{*{20}c} j \\ 0 \\ j \\ 0 \\ \end{array} } \right],\left[ {\begin{array}{*{20}c} j \\ 0 \\ 0 \\ j \\ \end{array} } \right],\left[ {\begin{array}{*{20}c} 0 \\ j \\ j \\ 0 \\ \end{array} } \right],\left[ {\begin{array}{*{20}c} 0 \\ j \\ 0 \\ j \\ \end{array} } \right]} \right\},$$$$\Upsilon _{4} = \left\{ {\left[ {\begin{array}{*{20}c} j \\ 0 \\ 0 \\ 0 \\ \end{array} } \right],\left[ {\begin{array}{*{20}c} 0 \\ j \\ 0 \\ 0 \\ \end{array} } \right],\left[ {\begin{array}{*{20}c} 0 \\ 0 \\ j \\ 0 \\ \end{array} } \right],\left[ {\begin{array}{*{20}c} 0 \\ 0 \\ 0 \\ j \\ \end{array} } \right]} \right\},$$ where ”0” denotes the un-activated states of the corresponding TA. Consequently, according to the formula of Eq. (1), the TSVs with the $$N_\textrm{t}=4$$ TAs are obtained in Table 1.

**Table 1:** EXAMPLES of forming the TSV symbol $\textbf{X}$ with the mapped 3D constellation symbol $s(m_x,m_y,m_z)$ or $\tilde{s}(\tilde{m}_x,\tilde{m}_y,\tilde{m}_z)$ and $N_t=4$.

$[{ b}_1,{ b}_2 ]$ bits		AI vectors from the set $\Gamma$	AI vector from the set $\Upsilon _{\alpha '}$	Real Vector of *X*	Imaginary Vector of *X*	TSV Symbol
${ b}_1$	${ b}_2$	$V_\alpha$	${\tilde{ V}}_\tau ^{\alpha '}$	${ X}^\Re$	${ X}^\Im$	*X*
0 0 0 0	0 0	$[1~0~0~0]^T$	$[j~0~j~0]^T$	$[m_x~0~0~0]^T$	$[jm_y~0~jm_z~0]^T$	$\left[ {m_x +jm_y,0,jm_z,0} \right] ^T$
0 0 0 1	0 1	$[0~1~0~0]^T$	$[j~0~0~j]^T$	$[0~m_x~0~0]^T$	$[jm_y~0~0~jm_z]^T$	$\left[ {jm_y,m_x,0,jm_z} \right] ^T$
0 0 1 0	1 0	$[0~0~1~0]^T$	$[0~j~j~0]^T$	$[0~0~m_x~0]^T$	$[0~jm_y~jm_z~0]^T$	$\left[ {0,jm_y,m_x+jm_z,0} \right] ^T$
0 0 1 1	1 1	$[0~0~0~1]^T$	$[0~j~0~j]^T$	$[0~0~0~m_x]^T$	$[0~jm_y~0~ jm_z]^T$	$\left[ {0,jm_y,0,m_x+jm_z} \right] ^T$
0 1 0 0	0 0	$[1~0~1~0]^T$	$[j~0~0~0]^T$	$[m_x~0~m_y~0]^T$	$[jm_z~0~0~0]^T$	$\left[ {m_x+jm_z,0, m_y,0} \right] ^T$
0 1 0 1	0 1	$[1~0~0~1]^T$	$[0~j~0~0]^T$	$[m_x~0~0~m_y]^T$	$[0~jm_z~0~0]^T$	$\left[ {m_x~jm_z~0~m_y} \right] ^T$
0 1 1 0	1 0	$[0~1~1~0]^T$	$[0~0~j~0]^T$	$[0 ~m_x~m_y~0]^T$	$[0~0~jm_z~0]^T$	$\left[ {0,m_x,jm_z+m_y,0} \right] ^T$
0 1 1 1	1 1	$[0~1~0~1]^T$	$[0~0~0~j]^T$	$[0 ~m_x~0~m_y]^T$	$[0~0~0 ~jm_z]^T$	$\left[ 0,m_x,0, m_y+jm_z \right] ^T$
1 0 0 0	0 0	$[1~1~0~0]^T$	$[j~0~0~0]^T$	$[m_x~m_y~ 0~0]^T$	$[jm_z~0~0~0]^T$	$\left[ {m_x +jm_z,m_y,0,0} \right] ^T$
1 0 0 1	0 1	$[0~0~1~1]^T$	$[0~j~0~0]^T$	$[0~0~m_x~m_y]^T$	$[0~jm_z~0~0]^T$	$\left[ {0,jm_z,m_x,m_y} \right] ^T$
1 0 1 0	1 0	$[1~0~0~0]^T$	$[0~j~j~0]^T$	$[{\tilde{m}_x}~0~0~0]^T$	$[0~j{\tilde{m}_y}~j{\tilde{m}_z}~0]^T$	$\left[ {{\tilde{m}_x},j{\tilde{m}_y},j{\tilde{m}_z},0} \right] ^T$
1 0 1 1	1 1	$[0~1~0~0]^T$	$[0~j~0~j]^T$	$[0~{\tilde{m}_x}~0~0]^T$	$[0~j{\tilde{m}_y}~0~ j{\tilde{m}_z}]^T$	$\left[ {0,{\tilde{m}_x}+j{\tilde{m}_y},0,j{\tilde{m}_z}} \right] ^T$
1 1 0 0	0 0	$[0~0~1~0]^T$	$[j~0~j~0]^T$	$[0~0~{\tilde{m}_x}~0]^T$	$[j{\tilde{m}_y}~0~j{\tilde{m}_z}~0]^T$	$\left[ {j{\tilde{m}_y},0,{\tilde{m}_x}+j{\tilde{m}_z},0} \right] ^T$
1 1 0 1	0 1	$[0~0~0~1]^T$	$[j~0~0~j]^T$	$[0~0~0~{\tilde{m}_x}]^T$	$[j{\tilde{m}_y}~0~0~jm_z]^T$	$\left[ {j{\tilde{m}_y},0,0,{\tilde{m}_x}+j{\tilde{m}_z}} \right] ^T$
1 1 1 0	1 0	$[1~0~1~0]^T$	$[0~0~j~0]^T$	$[{\tilde{m}_x}~0~{\tilde{m}_y}~ 0]^T$	$[0~0~j{\tilde{m}_z}~0]^T$	$\left[ {{\tilde{m}_x},0,{\tilde{m}_y}+j{\tilde{m}_z},0} \right] ^T$
1 1 1 1	1 1	$[1~0~0~1]^T$	$[0~0~0~j]^T$	$[{\tilde{m}_x}~0~0~{\tilde{m}_y}]^T$	$[0~0~0 ~j{\tilde{m}_z}]^T$	$\left[ {\tilde{m}_x},0,0,{\tilde{m}_y}+j{\tilde{m}_z} \right] ^T$

On the basis of the above design, three blocks of $[\mathbf{b_1}, \mathbf{b_2}, \mathbf{b_3}]$ information bits are modulated into one TSV symbol $\textbf{X}$ by using AI Vector Selectors and 3D Signal Mapper-$\textbf{I}$,$\textbf{II}$. In other words, the SE of the proposed SM-EIV-TDC is $\mathbf{b_1}+\mathbf{b_2}+\mathbf{b_3}$ bits/s/Hz, which can be expressed by

2 $$\begin{aligned} \eta = \textbf{b}_1 + \textbf{b}_2 + \textbf{b}_3 = \log _2 \left| \Gamma \right| + \log _2 N_\textrm{t} + \log _2 M. ~~(\mathrm {bits/s/Hz}) \end{aligned}$$

### Receiver with maximum likelihood detector

After that the TSV $\textbf{X}$ is transmitted over the MIMO Rayleigh fading channel matrix $\textbf{H}$, and adding the complex Gaussian white noise vector $\textbf{n}$, according to the formula of Eq. (1), the received signal vector at the receiver for the proposed SM-EIV-T3DC can be given by

3 $$\begin{aligned} \begin{array}{l} \textbf{y} = \mu \textbf{HX} + \textbf{n} \\ \begin{array}{*{20}c} {} \\ \end{array}\begin{array}{*{20}c} = \\ \end{array}\mu \cdot \textbf{HX}^\Re + \mu \cdot \textbf{HX}^\Im + \textbf{n} \\ \begin{array}{*{20}c} {} \\ \end{array} = \left\{ \begin{array}{l} \mu \cdot \textbf{H} \cdot \textrm{Mod}(\textbf{V}_\alpha ,m_x ) + \mu \cdot \textbf{H} \cdot \textrm{Mod}({\tilde{\textbf{V}}}_\tau ^1 ,\{ m_y ,m_z \} ) + \textbf{n},\begin{array}{*{20}c} {} \\ \end{array}1 \le \alpha \le \left| {\Gamma _1 } \right| \\ \mu \cdot \textbf{H} \cdot \textrm{Mod}(\textbf{V}_\alpha ,\{ m_x ,m_y \} ) + \mu \cdot \textbf{H} \cdot \textrm{Mod}({\tilde{\textbf{V}}}_\tau ^2 ,m_z ) + \textbf{n},\begin{array}{*{20}c} {} \\ \end{array}\left| {\Gamma _1 } \right| + 1 \le \alpha \le \left| {\Gamma _1 } \right| + \left| {\Gamma _2 } \right| \\ \mu \cdot \textbf{H} \cdot \textrm{Mod}(\textbf{V}_\alpha ,\tilde{m}_x ) + \mu \cdot \textbf{H} \cdot \textrm{Mod}({\tilde{\textbf{V}}}_\tau ^3 ,\{ \tilde{m}_y ,\tilde{m}_z \} ) + \textbf{n},\begin{array}{*{20}c} {} \\ \end{array}\left| {\Gamma _1 } \right| + \left| {\Gamma _2 } \right| + 1 \le \alpha \le \left| {\Gamma _1 } \right| + \left| {\Gamma _2 } \right| + \left| {\Gamma _3 } \right| \\ \mu \cdot \textbf{H} \cdot \textrm{Mod}(\textbf{V}_\alpha ,\{ \tilde{m}_x ,\tilde{m}_y \} ) + \mu \cdot \textbf{H} \cdot \textrm{Mod}({\tilde{\textbf{V}}}_\tau ^4 ,\tilde{m}_z ) + \textbf{n},\begin{array}{*{20}c} {} \\ \end{array}\left| {\Gamma _1 } \right| + \left| {\Gamma _2 } \right| + \left| {\Gamma _3 } \right| + 1 \le \alpha \le \left| {\Gamma } \right| \\ \end{array} \right. \\ \begin{array}{*{20}c} {} \\ \end{array} = \left\{ \begin{array}{l} \mu \cdot \textbf{h}_\alpha \cdot m_x + \mu \cdot (\textbf{h}_{\tau _1 } \cdot m_y \mathrm{+ } \textbf{h}_{\tau _2 } \cdot m_z ) + \textbf{n},\begin{array}{*{20}c} {} & {} \\ \end{array}1 \le \alpha \le \left| {\Gamma _1 } \right| \\ \mu \cdot (\textbf{h}_{\alpha _1 } \cdot m_x + \textbf{h}_{\alpha _2 } \cdot m_y \mathrm{) + }\mu \cdot \textbf{h}_\tau \cdot m_z + \textbf{n},\begin{array}{*{20}c} {} & {} \\ \end{array}\left| {\Gamma _1 } \right| + 1 \le \alpha \le \left| {\Gamma _1 } \right| + \left| {\Gamma _2 } \right| \\ \mu \cdot \textbf{h}_\alpha \cdot \tilde{m}_x + \mu \cdot (\textbf{h}_{\tau _1 } \cdot \tilde{m}_y \mathrm{+ } \textbf{h}_{\tau _2 } \cdot \tilde{m}_z ) + \textbf{n},\begin{array}{*{20}c} {} & {} \\ \end{array}\left| {\Gamma _1 } \right| + \left| {\Gamma _2 } \right| + 1 \le \alpha \le \left| {\Gamma _1 } \right| + \left| {\Gamma _2 } \right| + \left| {\Gamma _3 } \right| \\ \mu \cdot (\textbf{h}_{\alpha _1 } \cdot \tilde{m}_x + \textbf{h}_{\alpha _2 } \cdot \tilde{m}_y \mathrm{) + }\mu \cdot \textbf{h}_\tau \cdot \tilde{m}_z + \textbf{n},\begin{array}{*{20}c} {} & {} \\ \end{array}\left| {\Gamma _1 } \right| + \left| {\Gamma _2 } \right| + \left| {\Gamma _3 } \right| + 1 \le \alpha \le \left| {\Gamma } \right| \\ \end{array} \right. , \\ \end{array} \end{aligned}$$

where $\mu$ is a normalized factor and equaling to $\frac{1}{{E_{\textrm{av}} }},E_{\textrm{av}} = \frac{{E_{\textrm{av}}^\textrm{Q} + E_{\textrm{av}}^{\textrm{SQ}} }}{\textrm{2}}$, $E_{\textrm{av}}^\textrm{Q}$ and $E_{\textrm{av}}^{\textrm{SQ}}$ denote the average energy per 3D signal CP and secondary 3D signal CP, respectively. Mod($\cdot$) denotes the operation of modulating. Note that, the fading channel matrix $\textbf{H}$ is a matrix of complex values with $N_\textrm{r} \times N_\textrm{t}$ dimensions, each entry of which obeys the independent and identically distribution and is a complex Gaussian variable with *CN*(0, 1). the complex Gaussian white noise vector $\textbf{n} \in C^{N_\textrm{r} \times 1}$ with $CN(0,\sigma ^2 \textbf{I}_{N_\textrm{r }} )$, each entry of which obeys the independent and identically distribution.

On the basis of the above design, three blocks of $[\mathbf{b_1}, \mathbf{b_2}, \mathbf{b_3}]$ are respectively used for selecting an AI vector $\textbf{V}_\alpha$ from an AI vector set $\Gamma = \left\{ {\Gamma _1 ,\Gamma _2 ,\Gamma _3 ,\Gamma _4 } \right\}$, specifying one AI vector ${\tilde{\textbf{V}}}_\tau ^{\alpha '}$ from the set $\Upsilon _{\alpha '}$ and being mapped into one 3D signal CP. Furthermore, with the assumption of the perfectly known channel state information (CSI), the ML detector at the receiver is used to detect the receive signal vector $\textbf{y}$ for retrieving the original information bits of three blocks: $[\mathbf{b_1}, \mathbf{b_2}, \mathbf{b_3}]$. Hence, with using the ML detective algorithm, two AI vector indexes: $\alpha$ and $\tau$, the modulation order of *M* are jointly detected for the proposed SM-EIV-T3DC can be expressed as

4 $$\begin{aligned} \left[ {\hat{\alpha } ,\hat{\tau },\hat{M}} \right] = \arg \mathop {\min }\limits _{\alpha ,\tau , M} \left\| {\textbf{y} - \textbf{H} \cdot \mu \cdot \textbf{X}} \right\| ^2 \end{aligned}$$

where $\left\| \cdot \right\| ^2$ is the operation of Frobenius norm, $\hat{\alpha }$ is the estimate of $\alpha$ for the AI vector indexes in the set $\Gamma$. $\hat{\tau }$ is the estimate of $\tau$ for the AI vector indexes in the set $\Upsilon _{\alpha '}$. $\hat{M}$ is the estimate of *M* for the modulation order of the 3D signal constellation ($\Omega _\textrm{I}$ or $\Omega _\textrm{II}$).

## Methods design

### Design of the AI vector set $\Gamma$

The design objective of the set $\Gamma$ is to further expand the size of signal space for carrying more additional information bits, with the employing of two types of signal constellations, the AI vector set $\Gamma$ placed in the AI Vector Selector can be designed as

5 $$\begin{aligned} \Gamma = \left\{ {\underbrace{\textbf{V}_1 ,\textbf{V}_2 , \cdots ,\textbf{V}_{\left| {\Gamma _1 } \right| } }_{\Gamma _1 },\underbrace{\textbf{V}_{\left| {\Gamma _1 } \right| + 1} , \cdots ,\textbf{V}_{\left| {\Gamma _1 } \right| + \left| {\Gamma _2 } \right| } }_{\Gamma _2 },\underbrace{\textbf{V}_{\left| {\Gamma _1 } \right| + \left| {\Gamma _2 } \right| + 1} , \cdots ,\textbf{V}_{\left| {\Gamma _1 } \right| + \left| {\Gamma _2 } \right| + \left| {\Gamma _3 } \right| } }_{\Gamma _3 },\underbrace{\textbf{V}_{\left| {\Gamma _1 } \right| + \left| {\Gamma _2 } \right| + \left| {\Gamma _3 } \right| + 1} , \cdots ,\textbf{V}_{\left| \Gamma \right| } }_{\Gamma _4 }} \right\} , \end{aligned}$$

where $\left| {\Gamma _{\alpha '}} \right| , \alpha ' \in \{1,2,3,4\}$ is the number of the AI vectors in the set $\Gamma _\alpha '$, and all vectors in two subsets of both $\Gamma _1$ and $\Gamma _3$ are from the column vectors of an identity matrix $I_{N_\textrm{t}}$ with $N_\textrm{t} \times N_\textrm{t}$-dimensions. Nevertheless, all vectors in two subsets of both $\Gamma _2$ and $\Gamma _4$ are from the vector set $\Delta$ constructed by all possible vectors with the combinations of two AI indexes. Note that, $\left| {\Gamma _1 } \right|$ and $\left| {\Gamma _3 } \right|$ are equaling to the number of transmit antennas $N_\textrm{t}$ that satisfies the power of two, i.e., $\left| {\Gamma _1 } \right|$ =$\left| {\Gamma _3 } \right| =2^{\left\lfloor {\log _2 N_\textrm{t} } \right\rfloor }$, $\left| {\Gamma _2 } \right|$ is equaling to $C_{N_\textrm{t} }^2$, it is worth noting that $\left| {\Gamma _4 } \right|$ may be equaling or less than $C_{N_\textrm{t} }^2$ due to that $\left| {\Gamma } \right|$ needs to satisfy the power of two, i.e., $\left| {\Gamma _4 } \right| = \left| \Gamma \right| - (\left| {\Gamma _1 } \right| + \left| {\Gamma _2 } \right| + \left| {\Gamma _3 } \right| )$. For more intuitive explanation, assumed that $N_\textrm{t}=4$, the AI vector set $\Gamma$ can be expressed as

6 $$\begin{aligned} \Gamma = \left\{ {\underbrace{\begin{array}{*{20}c} 1 & 0 & 0 & 0 \\ 0 & 1 & 0 & 0 \\ 0 & 0 & 1 & 0 \\ 0 & 0 & 0 & 1 \\ \end{array}}_{\Gamma _1 }\,\underbrace{\begin{array}{*{20}c} 1 & 1 & 0 & 0 & 1 & 0 \\ 0 & 0 & 1 & 1 & 1 & 0 \\ 1 & 0 & 1 & 0 & 0 & 1 \\ 0 & 1 & 0 & 1 & 0 & 1 \\ \end{array}}_{\Gamma _2 }\,\underbrace{\begin{array}{*{20}c} 1 & 0 & 0 & 0 \\ 0 & 1 & 0 & 0 \\ 0 & 0 & 1 & 0 \\ 0 & 0 & 0 & 1 \\ \end{array}}_{\Gamma _3 }\,\underbrace{\begin{array}{*{20}c} 1 & 1 \\ 0 & 0 \\ 1 & 0 \\ 0 & 1 \\ \end{array}}_{\Gamma _4 }} \right\} . \end{aligned}$$

From Eq. (6), it can be seen that the AI vector set $\Gamma _4$ only contains two columns of vectors. Obviously, the design of the set $\Gamma$ expands the size of signal spaces in comparisons with the classic ESM scheme.

### Design of the AI vector sets: $\{\Upsilon _1$, $\Upsilon _2$, $\Upsilon _3$, $\Upsilon _4\}$

In order to further exploit the additional information bits, four AI vector sets: {$\Upsilon _1$, $\Upsilon _2$, $\Upsilon _3$, $\Upsilon _4$} are designed. Moreover, to avoid generating the same TSVs, two types of 3D CPs are employed, the $C_{N_\textrm{t} }^1 + C_{N_\textrm{t} }^2$ number of AI vectors in the AI vector set $\Gamma$ is used to modulate the C3D signal CPs and the remaining AI vectors in the set $\Gamma$ is used to modulate the modified S3D signal CPs.

The design of the auxiliary AI vector sets $\{\Upsilon _1$, $\Upsilon _2$, $\Upsilon _3$, $\Upsilon _4\}$ is architected based on three foundational principles to ensure optimal performance:

- **Complementarity:** The structure of each $\Upsilon _{\alpha '}$ is directly complementary to its counterpart $\Gamma _{\alpha '}$. Specifically, when the selected vector from $\Gamma _{\alpha '}$ has *k* non-zero elements (modulating *k* components of the 3D symbol), the corresponding vector from $\Upsilon _{\alpha '}$ is designed to have $3-k$ non-zero elements (modulating the remaining components). This guarantees that every TSV consistently utilizes three active antennas.
- **Orthogonality and domain expansion:** All non-zero elements in the $\Upsilon$ sets are assigned the imaginary unit *j*. This creates a clear separation between the in-phase (real) and quadrature (imaginary) spatial dimensions, expanding the index domain orthogonally and preventing ambiguity between different TSVs.
- **Constellation association for MED enhancement:** The pairing of $\Upsilon _1, \Upsilon _2$ with the C3D constellation and $\Upsilon _3, \Upsilon _4$ with the MS3D constellation provides a structured mapping. This allows for joint optimization of the signal constellations to maximize the squared MED between TSVs, as detailed in Sect. Design of the 3D constellations.

Based on these principles, the construction rules for the four sets are defined as follows:

1. If the selected AI vector $\textbf{V}_\alpha$ is from the vector subset $\Gamma _1$ ( i.e., $\alpha \le \left| {\Gamma _1 } \right|$), the AI vector set $\Upsilon _1$ may be designed as $\Upsilon _1 = \left\{ {{\tilde{\textbf{V}}}_1^1 ,{\tilde{\textbf{V}}}_2^1 , \cdots ,{\tilde{\textbf{V}}}_{N_\textrm{t}} ^1 } \right\}$ from the vector set $\tilde{\Delta }$ whose vectors have two non-zero elements equaling to $''j''$, i.e., ${\tilde{\textbf{V}}}_\tau ^1 \in {\tilde{\Delta }}, \tau \in \{1,2,\cdots ,N_\textrm{t}\}$. This design, complementary to the single-active-element structure of $\Gamma _1$, ensures that the resultant spatial vector $\textbf{S}^1=\textbf{V}_\alpha +{\tilde{\textbf{V}}}_\tau ^1$ by performing the Adder in Fig. 1 has exactly three non-zero elements for modulating the three components of the 3D symbol.
2. If the selected AI vector $\textbf{V}_\alpha$ is from the vector subset $\Gamma _2$ ( i.e., $\left| {\Gamma _1 } \right| +1\le \alpha \le \left| {\Gamma _1 } \right| +\left| {\Gamma _2 } \right|$), the AI vector set $\Upsilon _2$ can be designed as $\Upsilon _2 = \left\{ {{\tilde{\textbf{V}}}_1^2 ,{\tilde{\textbf{V}}}_2^2 , \cdots ,{\tilde{\textbf{V}}}_{N_\textrm{t} }^2 } \right\}$ from one identity matrix $\tilde{I}_{N_\textrm{t}}$ whose vectors have one non-zero element with the imaginary sign ”*j*”,i.e., ${\tilde{\textbf{V}}}_\tau ^2 \in {\textbf{I}}_{N_\textrm{t}}, \tau \in \{1,2,\cdots ,N_\textrm{t}\}$. Consequently, the resulted spatial vector $\textbf{S}^2=\textbf{V}_\alpha +{\tilde{\textbf{V}}}_\tau ^2$ has three non-zero elements for modulating one 3D signal CP.
3. Similarly, if the selected AI vector $\mathbf{V_\alpha } \in \Gamma _3$, i.e., $\left| {\Gamma _1 } \right| +\left| {\Gamma _2 } \right| +1\le \alpha \le \left| {\Gamma _1 } \right| +\left| {\Gamma _2 } \right| +\left| {\Gamma _3 } \right|$. In order to be capable of modulating three components: $m_x, m_y, m_z$, the AI vector set $\Upsilon _3$ can be designed as $\Upsilon _3 = \Upsilon _1$. Such that, the resulted spatial vector $\textbf{S}^3=\textbf{V}_\alpha +{\tilde{\textbf{V}}}_\tau ^3$ has also three non-zero elements for modulating one 3D signal CP.
4. Also, if the selected AI vector $\mathbf{V_\alpha } \in \Gamma _4$, i.e., $\left| {\Gamma _1 } \right| +\left| {\Gamma _2 } \right| +\left| {\Gamma _3 } \right| +1\le \alpha \le \left| {\Gamma } \right|$. The AI vector set $\Upsilon _4$ can be constructed by $\left| {\Gamma } \right| -(\left| {\Gamma _1 } \right| +\left| {\Gamma _2 } \right| +\left| {\Gamma _3 } \right| )$ vectors from the vector set $\Upsilon _2$. Such that, the obtained $\textbf{S}^4=\textbf{V}_\alpha +{\tilde{\textbf{V}}}_\tau ^4$ has also three non-zero elements to modulate one 3D signal CP.

In summary, the partitioned design of the sets $\{\Upsilon _1$, $\Upsilon _2$, $\Upsilon _3$, $\Upsilon _4\}$, combined with the use of two distinct 3D constellations, is a deliberate strategy to expand the spatial index domain while providing a structured framework for maximizing the MED, which is analytically and empirically verified in the following sections.

### Design of the 3D constellations

In this subsection, our design objective for 3D signal constellation is to maximize the squared MED between the normalized TSVs (e.g., multiple types of the TSVs (e.g., $\textbf{X}^1, \textbf{X}^2, \textbf{X}^3, \textbf{X}^4$) are obtained to be normalized).

Based on the above-mentioned design of the AI vector sets of both $\Gamma$ and $\Upsilon$, if we only employ one type of 3D signal constellation to be modulated by the selected vectors from two AI vector sets: $\Gamma$ and $\Upsilon$ in our proposed SM-EIV-T3DC, it will cause unrecoverable detection errors at the receiver. In order to avoid this problem, two types of 3D signal constellations are introduced, one of which is the C3D constellation, another one of which is obtained by constructing the secondary QAM constellation^6,7^, e.g., 8-ary 3D signal constellation: $\left( { \pm {\mathrm 2,} \pm {\mathrm 2,} \pm {\mathrm 2}} \right)$. Furthermore, the $C_{N_\textrm{t} }^1 + C_{N_\textrm{t} }^2$ number of AI vectors in the AI vector set $\Gamma$ are used to modulate the signal 3D CPs from the C3D constellation, as well as all AI vectors in both of {$\Upsilon _1$, $\Upsilon _2$}. The remaining AI vectors in the set $\Gamma$ are used to modulate the signal 3D CPs from the S3D constellation, as well as all AI vectors in both of {$\Upsilon _3$, $\Upsilon _4$}. Thus, two types of 3D signal constellations for 8, 16, 32, 64-ary 3D constellations may be provided in Table 2.

**Table 2:** EXAMPLEs of 8, 16, 32, 64-ary conventional and secondary 3D constellations.

Order of Signal CPs	Conventional TD constellation	Secondary TD constellation
8-ary	$\left( { \pm {\mathrm 1,} \pm {\mathrm 1,} \pm {\mathrm 1}} \right)$	$\left( { \pm {\mathrm 2,} \pm {\mathrm 2,} \pm {\mathrm 2}} \right)$
16-ary	$\left\{ {\left( { \pm {\mathrm 1,} \pm {\mathrm 1,} \pm {\mathrm 1}} \right) ,\left( { \pm {\mathrm 1,} \pm {\mathrm 1,} \pm {\mathrm 3}} \right) } \right\}$	$\left\{ {\left( { \pm {\mathrm 2,} \pm {\mathrm 2,} \pm {\mathrm 2}} \right) ,\left( { \pm {\mathrm 4,} \pm {\mathrm 2,} \pm {\mathrm 2}} \right) } \right\}$
32-ary	$$\left\{ \begin{array}{l} \left( { \pm {\mathrm 1,} \pm {\mathrm 1,} \pm {\mathrm 1}} \right) ,\left( { \pm {\mathrm 1,} \pm {\mathrm 1,} \pm {\mathrm 3}} \right) , \\ \left( { \pm {\mathrm 1,} \pm {\mathrm 3,} \pm {\mathrm 1}} \right) ,\left( { \pm {\mathrm 3,} \pm {\mathrm 1,} \pm {\mathrm 1}} \right) \\ \end{array} \right\}$$	$$\left\{ \begin{array}{l} \left( { \pm {\mathrm 2,} \pm {\mathrm 2,} \pm {\mathrm 2}} \right) ,\left( { \pm {\mathrm 4,} \pm {\mathrm 2,} \pm {\mathrm 2}} \right) , \\ \left( { \pm {\mathrm 2,} \pm {\mathrm 4,} \pm {\mathrm 2}} \right) ,\left( { \pm {\mathrm 2,} \pm {\mathrm 2,} \pm {\mathrm 4}} \right) \\ \end{array} \right\}$$
64-ary	$$\left\{ \begin{array}{l} \left( { \pm {\mathrm 1,} \pm {\mathrm 1,} \pm \textrm{1}} \right) ,\left( { \pm \textrm{1,} \pm \textrm{1,} \pm \textrm{3}} \right) , \\ \left( { \pm \textrm{1,} \pm \textrm{3,} \pm \textrm{1}} \right) ,\left( { \pm \textrm{3,} \pm \textrm{1,} \pm \textrm{1}} \right) , \\ \left( { \pm \textrm{3,} \pm \textrm{3,} \pm \textrm{1}} \right) ,\left( { \pm \textrm{3,} \pm \textrm{1,} \pm \textrm{3}} \right) , \\ \left( { \pm \textrm{1,} \pm \textrm{3,} \pm \textrm{3}} \right) ,\left( { \pm \textrm{3,} \pm \textrm{3,} \pm \textrm{3}} \right) \\ \end{array} \right\}$$	$$\left\{ \begin{array}{l} \left( { \pm {\mathrm 2,} \pm {\mathrm 2,} \pm \textrm{2}} \right) ,\left( { \pm \textrm{4,} \pm \textrm{2,} \pm \textrm{2}} \right) , \\ \left( { \pm \textrm{2,} \pm \textrm{4,} \pm \textrm{2}} \right) ,\left( { \pm \textrm{2,} \pm \textrm{2,} \pm \textrm{4}} \right) , \\ \left( { \pm \textrm{4,} \pm \textrm{4,} \pm \textrm{2}} \right) ,\left( { \pm \textrm{4,} \pm \textrm{2,} \pm \textrm{4}} \right) , \\ \left( { \pm \textrm{2,} \pm \textrm{4,} \pm \textrm{4}} \right) ,\left( { \pm \textrm{2,} \pm \textrm{2,} \pm \textrm{6}} \right) \\ \end{array} \right\}$$

Based on the analysis of the squared MED in conventional MIMO-IM systems, such as the QSM, ESIM and JPGA-ISM, it can be seen that the squared MED between the normalized TSVs: $\textbf{X}$ is given by

7 $$\begin{aligned} d_{\min ,{ \bar{ \textbf{X}}}}^2 \mathop = \limits _{{\bar{ \textbf{X}}} \ne { {\hat{\bar{ \textbf{X}}}}}} \min \left\| {{\bar{ \textbf{X}}} - { {{\hat{\bar{ \textbf{X}}}}}}} \right\| ^2 = \frac{2}{{E_\textrm{av}}}, \end{aligned}$$

where $E_\textrm{av}$ is the average energy per TSV. From this, based on the consideration of the squared MED in Eq. (7), the design requirement of the squared MED for the proposed SM-EIV-T3DC may be given by $\frac{2}{{E\textrm{av}}}$. Hence, the secondary 3D signal constellations for 8, 16, 32, 64-ary 3D constellations may be modified and designed for maximizing the squared MED, as shown in the right column of Table 3. Consequently, the C3D signal constellations are combined with the modified secondary 3D (MS3D) signal constellations for the proposed SM-EIV-T3DC, as shown in Table 3.

**Table 3:** EXAMPLEs of 8, 16, 32, 64-ary conventional and modified secondary 3D constellations.

Order of signal CPs	Conventional TD constellation	Modified secondary TD constellation
8-ary	$\left( { \pm {\mathrm 1,} \pm {\mathrm 1,} \pm {\mathrm 1}} \right)$	$\left( { \pm {\mathrm 1,} \pm {\mathrm 2,} \pm {\mathrm 2}} \right)$
16-ary	$\left\{ {\left( { \pm {\mathrm 1,} \pm {\mathrm 1,} \pm {\mathrm 1}} \right) ,\left( { \pm {\mathrm 1,} \pm {\mathrm 1,} \pm {\mathrm 3}} \right) } \right\}$	$\left\{ {\left( { \pm {\mathrm 1,} \pm {\mathrm 2,} \pm {\mathrm 2}} \right) ,\left( { \pm {\mathrm 2,} \pm {\mathrm 1,} \pm {\mathrm 2}} \right) } \right\}$
32-ary	$$\left\{ \begin{array}{l}\left( { \pm {\mathrm 1,} \pm {\mathrm 1,} \pm {\mathrm 1}} \right) ,\left( { \pm {\mathrm 1,} \pm {\mathrm 1,} \pm {\mathrm 3}} \right) , \\ \left( { \pm {\mathrm 1,} \pm {\mathrm 3,} \pm {\mathrm 1}} \right) ,\left( { \pm {\mathrm 3,} \pm {\mathrm 1,} \pm {\mathrm 1}} \right) \\ \end{array} \right\}$$	$$\left\{ \begin{array}{l}\left( { \pm {\mathrm 1,} \pm {\mathrm 2,} \pm {\mathrm 2}} \right) ,\left( { \pm {\mathrm 2,} \pm {\mathrm 1,} \pm {\mathrm 2}} \right) , \\ \left( { \pm {\mathrm 2,} \pm {\mathrm 2,} \pm {\mathrm 1}} \right) ,\left( { \pm {\mathrm 3,} \pm {\mathrm 2,} \pm {\mathrm 2}} \right) \\ \end{array} \right\}$$
64-ary	$$\left\{ \begin{array}{l}\left( { \pm {\mathrm 1,} \pm {\mathrm 3,} \pm \textrm{1}} \right) ,\left( { \pm \textrm{1,} \pm \textrm{1,} \pm \textrm{3}} \right) , \\ \left( { \pm \textrm{1,} \pm \textrm{3,} \pm \textrm{1}} \right) ,\left( { \pm \textrm{3,} \pm \textrm{1,} \pm \textrm{1}} \right) , \\ \left( { \pm \textrm{3,} \pm \textrm{3,} \pm \textrm{1}} \right) ,\left( { \pm \textrm{3,} \pm \textrm{1,} \pm \textrm{3}} \right) , \\ \left( { \pm \textrm{1,} \pm \textrm{3,} \pm \textrm{3}} \right) ,\left( { \pm \textrm{3,} \pm \textrm{3,} \pm \textrm{3}} \right) \\ \end{array} \right\}$$	$$\left\{ \begin{array}{l}\left( { \pm {\mathrm 1,} \pm {\mathrm 2,} \pm \textrm{2}} \right) ,\left( { \pm \textrm{2,} \pm \textrm{1,} \pm \textrm{2}} \right) , \\ \left( { \pm \textrm{2,} \pm \textrm{2,} \pm \textrm{1}} \right) ,\left( { \pm \textrm{3,} \pm \textrm{2,} \pm \textrm{2}} \right) , \\ \left( { \pm \textrm{2,} \pm \textrm{3,} \pm \textrm{2}} \right) ,\left( { \pm \textrm{2,} \pm \textrm{2,} \pm \textrm{3}} \right) , \\ \left( { \pm \textrm{4,} \pm \textrm{2,} \pm \textrm{1}} \right) ,\left( { \pm \textrm{4,} \pm \textrm{1,} \pm \textrm{2}} \right) \\ \end{array} \right\}$$

Hence, based on the above-mentioned 3D signal constellation design, we make a comparison of the squared MED for verifying the advantage of the MS3D constellation. Here, in order to intuitively describe the SM-EIV-T3DC with different combinations of two 3D constellations, the conventional 3D (C3D) constellation and secondary 3D (S3D) constellation (as shown in Table 2), and the C3D constellation and modified S3D (MS3D) constellation (as shown in Table 3) are abbreviated as C3D-S3D and C3D-MS3D constellations, respectively. The squared MEDs can be calculated by the Eq. (7). Consequently, the squared MED $d_{\min ,{\bar{ \textbf{X}}}}^2$ of the SM-EIV-T3DC with 8, 16, 32, 64-ary C3D-S3D constellations are 2/7.5, 2/9.5, 2/15, 2/29.5, respectively. Similarly, in Table 3, the squared MED $d_{\min ,{\bar{ \textbf{X}}}}^2$ of the SM-EIV-T3DC with 8, 16, 32, 64-ary C3D-MS3D constellations are 2/6, 2/8, 2/10, 2/15. Obviously, the SM-EIV-T3DC with 8, 16, 32, 64-ary C3D-MS3D constellations has the advantage of the squared MED.

**Fig. 2:**
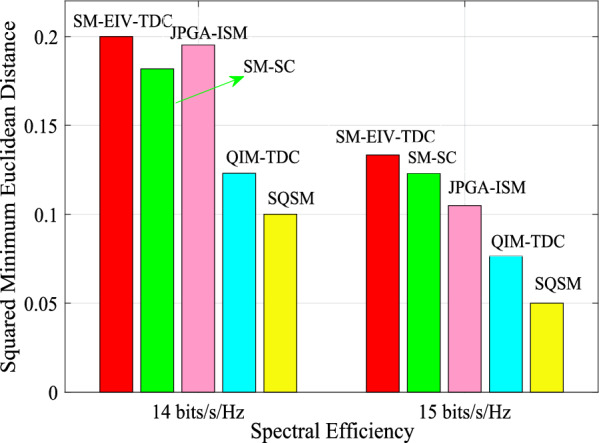
The squared MED comparisons of the SM-EIV-T3DC with the SM-SC, JPGA-ISM, QIM-TDC and SQSM schemes at the SEs of 14, 15 bits/s/Hz with $\{N_\textrm{t},N_\textrm{r}\}=\{8,8\}$.

To further outstand the advantage of the proposed SM-EIV-T3DC as compared with various schemes at the different SEs, according to the calculation of Eq. (7), the squared MEDs of the SM-EIV-T3DC are provided and compared with various schemes such as the QIM-TDC, SM-SC, JPGA-SIM and SQSM schemes at the SEs of {14, 15} bits/s/Hz with $\{N_\textrm{t},N_\textrm{r}\}=\{8,8\}$, as shown in Fig. 2. Note that, at the SEs of {14, 15} bits/s/HZ, the squared MEDs of the SM-EIV-T3DC using {32, 64}-ary C3D-MS3D constellations are 2/10, 2/15, that of the SM-SC using {64, 128}-ary 3DCII are 2/11, 2/16.25, that of the JPGA-ISM using $\{D=3; (2,2,2), (2,2,4)\}$ are 2/10.2426, 2/19.0711, that of the QIM-TDC using {128, 256}-ary 3DCII are 2/16.25, 2/26.125, and that of the SQSM using {64, 128}-ary QAM are 2/42, 2/82, respectively.

## Performance analysis

### Computational complexity

In this subsection, the computational complexity of the proposed SM-EIV-T3DC scheme is analyzed and compared with several existing MIMO-IM schemes, including JPGA-ISM, SM-SC, and QIM-TDC. The complexity is evaluated in terms of the number of real multiplication required by the ML detector at the receiver, which is widely used as a benchmark for performance comparison in MIMO-IM systems.

For fair comparison, the JPGA-ISM and SM-SC schemes are discussed based on the case of $D=3$^30,32^ active transmit antennas, which aligns with the configuration of the proposed SM-EIV-T3DC. In the SM-EIV-T3DC, JPGA-ISM, SM-SC, and QIM-TDC schemes, since each transmitted vector contains only three non-zero elements, they have the same number of real multiplications per candidate vector when using the ML algorithm as expressed in Eq. (4). The calculation process can be summarized in the following steps: Firstly, for the calculation of $\textbf{H} \cdot {\bar{ \textbf{X}}}$, the SM-EIV-T3DC, JPGA-ISM, SM-SC, and QIM-TDC schemes require $6 \cdot N_\textrm{r}$ real multiplications for each candidate vector, where ${\bar{ \textbf{X}}} = \mu \textbf{X}$ is the normalized transmitted vector. Then, it requires $4N_\textrm{r}$ real multiplications to calculate the square, i.e., Calculating $\left\| {\textbf{Y} - \textbf{H} \cdot {\bar{ \textbf{X}}}} \right\| ^2$. Consequently,

- QIM-TDC: $$\gamma _{\mathrm {SM-EIV-T3DC}}=10 \times N_{\mathrm r} \times 2^\eta$$
- JPGA-ISM: $$\gamma _{\mathrm {JPGA-ISM}}=10 \times N_{\mathrm r} \times 2^\eta$$
- SM-SC: $$\gamma _{\mathrm {SM-SC}}=10 \times N_{\mathrm r} \times 2^\eta$$
- QIM-TDC: $$\gamma _{\mathrm {QIM-TDC}}=10 \times N_{\mathrm r} \times 2^\eta$$.

where $\eta$ denotes the spectral efficiency.

Meanwhile, simulation results in the Sect. Simulation and numerical results demonstrate that the proposed SM-EIV-T3DC also achieves superior BER performance compared with the JPGA-ISM, SM-SC, and QIM-TDC schemes. Therefore, This makes the SM-EIV-T3DC an attractive solution for applications requiring both high reliability and spectral efficiency.

### Bit error probability

In this subsection, the theoretical upper bound of the average BEP is analyzed and derived. According to both Eq. (3) and Eq. (4), and the theoretical derivation in^33^, the conditional pairwise error probability (PEP) on the channel matrix $\textbf{H}$ can be derived by the following calculation, as follows:

8 $$\begin{aligned} \begin{array}{l} P\left( {\left. {{\bar{ \textbf{X}}} \rightarrow { \hat{\bar{ \textbf{X}}}}} \right| \textbf{H}} \right) = P\left( {\left. {\left\| {\textbf{y} - { H\bar{\textbf{X}}}} \right\| ^2> \left\| {\textbf{y} - { H\hat{\bar{ \textbf{X}}}}} \right\| ^2 } \right| \textbf{H}} \right) \\ ~~~~~~~~~~~~~~~~~~~~~~~~~~= P\left( {\left. {\left\| \textbf{n} \right\| ^2> \left\| {\textbf{H}({\bar{ \textbf{X}}} - { \hat{ \bar{ \textbf{X}}}}) + \textbf{n}} \right\| ^2 } \right| \textbf{H}} \right) \\ ~~~~~~~~~~~~~~~~~~~~~~~~~~= P\left( {\left. {\left\| \textbf{n} \right\| ^2> \left\| {\textbf{H}({\bar{ \textbf{X}}} - {\hat{\bar{ \textbf{X}}}})} \right\| ^2 + \left\| \textbf{n} \right\| ^2 + 2\Re \{ \textbf{n}^\chi \cdot \textbf{H}({\bar{ \textbf{X}}} - {\hat{\bar{ \textbf{X}}}}) \cdot \} } \right| \textbf{H}} \right) \\ ~~~~~~~~~~~~~~~~~~~~~~~~~~ = P\left( {\left. {2\Re \{ \textbf{n}^\chi \cdot \textbf{H}({\bar{ \textbf{X}}} - {\hat{\bar{ \textbf{X}}}})\} < \left\| {\textbf{H}({\bar{ \textbf{X}}} - {\hat{\bar{ \textbf{X}}}})} \right\| ^2 } \right| \textbf{H}} \right) \\ \end{array}, \end{aligned}$$

where $\{\cdot \}^\chi$ is the operation of the conjugate transpose, ${\bar{ \textbf{X}}} = \mu \textbf{X}$, and ${\hat{\bar{ \textbf{X}}}}$ is the estimate of ${\bar{ \textbf{X}}}$, $\Re \{ \textbf{n}^\chi \cdot \textbf{H}({\bar{ \textbf{X}}} - {\hat{\bar{ \textbf{X}}}})\}$ is the real part of $\textbf{n}^\chi \cdot \textbf{H}({\bar{ \textbf{X}}} - {\hat{\bar{ \textbf{X}}}})$ that is a complex variable with the cyclic symmetric complex Gaussian distribution. Furthermore, the variable of $\Re \{ \textbf{n}^\chi \cdot \textbf{H}({\bar{ \textbf{X}}} - {\hat{\bar{ \textbf{X}}}})\}$ obeys the zero mean and the variance of $N_0 = \frac{{\sigma ^2 }}{2}\left\| {\textbf{H}({\bar{ \textbf{X}}} - {\hat{\bar{ \textbf{X}}}})} \right\| ^2$, i.e., $CN(0, N_0)$. In order to transform the Eq. (8) into the form of the standard normal distribution, the variable of $\Re \{ \textbf{n}^\chi \cdot \textbf{H}({\bar{ \textbf{X}}} - {\hat{\bar{ \textbf{X}}}})\}$ is normalized as $\sqrt{N_0 }$. Meanwhile, utilizing the expression of the Gaussian *Q*-function^33^, the Eq. (8) for the conditional PEP can be written as

9 $$\begin{aligned} P\left( {\left. {{\bar{ \textbf{X}}} \rightarrow {\hat{\bar{ \textbf{X}}}}} \right| \textbf{H}} \right) = P\left( {\left. {2\sqrt{N_0 } Z< \left\| {\textbf{H}({\bar{ \textbf{X}}} - {\hat{\bar{ \textbf{X}}}})} \right\| ^2 } \right| \textbf{H}} \right) = P\left( {\left. {Z < \sqrt{\frac{{\left\| {\textbf{H}({\bar{ \textbf{X}}} - {\hat{\bar{ \textbf{X}}}})} \right\| ^2 }}{{2\sigma ^2 }}} } \right| \textbf{H}} \right) = Q\left( {\sqrt{\frac{{\left\| {\textbf{H}({\bar{ \textbf{X}}} - {\hat{\bar{ \textbf{X}}}})} \right\| ^2 }}{{2\sigma ^2 }}} } \right) , \end{aligned}$$

where *Z* is a normalized Gaussian random variable of $\Re \{ \textbf{n}^\chi \cdot \textbf{H}({\bar{ \textbf{X}}} - {\hat{\bar{ \textbf{X}}}})\}$.

Furthermore, in order to eliminate the channel matrix $\textbf{H}$ for the unconditional PEP $P\left( {{\bar{ \textbf{X}}} \rightarrow {\hat{\bar{ \textbf{X}}}}} \right)$, i.e., obtaining the average PEP. On the basis of $Q\left( x \right) = \frac{1}{\pi }\int _0^{\frac{\pi }{2}} {\exp \left( { - \frac{{x^2 }}{{2\sin ^2 \theta }}} \right) } d\theta$, the Eq. (9) is taken the expectation over the channel matrix $\textbf{H}$, thus the unconditional PEP can be expressed as

10 $$\begin{aligned} P\left( {{\bar{ \textbf{X}}} \rightarrow {\hat{\bar{ \textbf{X}}}}} \right) = E_\textbf{H} \left\{ {\frac{1}{\pi }\int _0^{\frac{\pi }{2}} {\exp \left( { - \frac{{\left\| {\textbf{H}({\bar{ \textbf{X}}} - {\hat{\bar{ \textbf{X}}}})} \right\| ^2 }}{{2\sigma ^2 \sin ^2 \theta }}} \right) } d\theta } \right\} = \int _0^\infty {\left[ {\frac{1}{\pi }\int _0^{\frac{\pi }{2}} {\exp \left( { - \frac{{\left\| {\textbf{H}({\bar{ \textbf{X}}} - {\hat{\bar{ \textbf{X}}}})} \right\| ^2 }}{{2\sigma ^2 \sin ^2 \theta }}} \right) } d\theta } \right] \cdot f(\varsigma )d\varsigma }, \end{aligned}$$

where $f(\varsigma )$ is the probability density function of the random variable $\varsigma = \frac{{\textbf{H}^\chi \textbf{H}}}{{\sqrt{\left\| {\textbf{H}^\chi \textbf{H}} \right\| ^2 } }}$ with the normalized chi-squared distribution.

After some mathematical calculus operations, and using the moment-generating function^33^, the closed-form of the average PEP is calculated as

11 $$\begin{aligned} P\left( {{\bar{ \textbf{X}}} \rightarrow {\hat{\bar{ \textbf{X}}}}} \right) = \frac{1}{\pi }\int _0^{{\pi 2}} {\left[ {\int _0^\infty {\exp \left( { - \frac{{\left\| {\textbf{H}({\bar{ \textbf{X}}} - {\hat{\bar{ \textbf{X}}}})} \right\| ^2 }}{{2\sigma ^2 \sin ^2 \theta }}} \right) } \cdot f(\varsigma )d\varsigma } \right] } d\theta = \left( {\frac{1}{2} - \frac{\textrm{A}}{2}} \right) ^{N_\textbf{r} } \sum \limits _{\omega = 0}^{N_r - 1} {\left( {\begin{array}{*{20}c} {N_\textrm{r} - 1 + \omega } \\ \omega \\ \end{array}} \right) \left( {\frac{1}{2} + \frac{\textrm{A}}{2}} \right) ^\omega }, \end{aligned}$$

where $\textrm{A} = \sqrt{\frac{{{{d_{\min ,{\bar{ \textbf{X}}}}^2 } {\sigma ^2 }}}}{{4 + {{d_{\min ,{\bar{ \textbf{X}}}}^2 } {\sigma ^2 }}}}}$, which is mainly determined by the ratio of the normalized squared MED between the TSVs to the variance of white noise $\textbf{n}$.

Consequently, by using the upper bound technique^33^, the average bit error probability may be given by

12 $$\begin{aligned} P_\textrm{e} = \frac{1}{{2^\eta }}\sum \limits _{{\bar{ \textbf{X}}}} {\sum \limits _{{\bar{ \textbf{X}}} \ne {\hat{\bar{ \textbf{X}}}}} {P\left( {{\bar{ \textbf{X}}} \rightarrow {\hat{\bar{ \textbf{X}}}}} \right) } } \cdot \frac{{d\left( {{\bar{ \textbf{X}}} \rightarrow { \hat{ \bar{ \textbf{X}}}}} \right) }}{\eta }, \end{aligned}$$

where $d\left( {{\bar{ \textbf{X}}} \rightarrow { \hat{ \bar{ \textbf{X}}}}} \right)$ is the erroneous bits between ${\bar{ \textbf{X}}}$ and ${ \hat{ \bar{ \textbf{X}}}}$, i.e., namely the hamming distance.

## Simulation and numerical results

### Simulation results under perfect channel state information

In this section, the performances for the BER versus SNR between the proposed SM-EIV-T3DC and the SM-SC, JPGA-ISM, V-BLAST, SQSM and QIM-TDC schemes are depicted and compared at the same configuration of both the different SEs (e.g., $\{11,~12,~13,~14,~15\}$ bits/s/Hz) and the RF number of TAs. In all simulations with the MATLAB software, the channel state information is assumed to be ideally known at the receiver, and the BER performances comparisons between these schemes are evaluated by using the Monte Carlo method with the ML detector. For the intuitively expression of the parameters of simulation results, *L*-3DCII is presented in Ref^19^. for the QIM-TDC and SM-SC schemes and $L'$-$L''$QAM denotes that $L'$-QAM and $L''$-QAM are employed simultaneously. Also, we define the JPGA-ISM with $\{D; (M^1 \cdots M^D)\}$, where $\{D; (M^1 \cdots M^D)\}$ denotes the employed *D* number of $M^1 \cdots M^D$-PAM or SPAM constellations (as is seen in Ref^32^.).

**Fig. 3:**
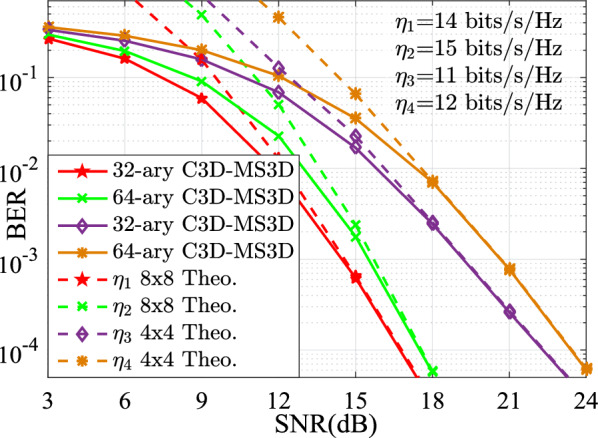
BER curves comparison of the simulation and theoretical results at $\{N_\textrm{t},N_\textrm{r}\}\in \{(4,4),(8,8)\}$.

In order to verify the feasibility and effectiveness of the proposed SM-EIV-T3DC, the theoretical results obtained by Eq. (12) and the simulation results are depicted in Fig. 3. Obviously, it can be observed that the simulation results are in agreement with the theoretical results in the high SNR region at different TAs and SEs (i.e., $\{(N_\textrm{t},N_\textrm{r})= (4,4), \{11,~12\} ~\mathrm{{bits/s/Hz}}\}$ and $\{(N_\textrm{t},N_\textrm{r})= (8,8), \{14,~15\}~\mathrm{{bits/s/Hz}}\}$) in the high SNR region.

**Fig. 4:**
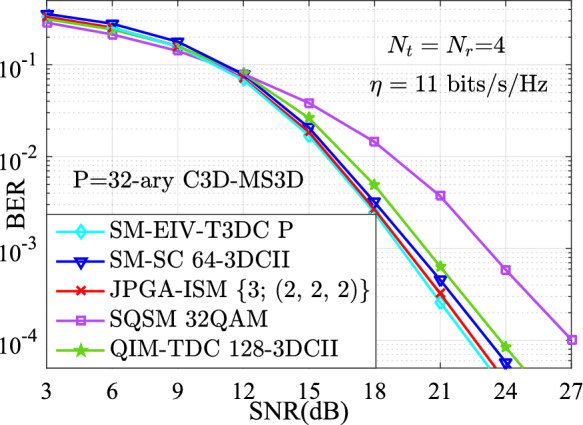
BER versus SNR for the SM-EIV-T3DC, SM-SC, JPGA-ISM, SQSM and QIM-TDC schemes with $\{N_\textrm{t},N_\textrm{r}\}=(4,4)$ at the SE of 11 bits/s/Hz.

Fig. 4, at the same SEs (i.e., $\eta = 11$ bits/s/Hz) and the same number of TAs (i.e., $N_\textrm{t}=N_\textrm{r}=4$), depicts the simulation results of the BER versus SNR for various schemes. Specifically, the SM-EIV-T3DC using 32-ary C3D-MS3D (i.e., 32-ary conventional 3D and 32-ary MS3D) is compared with the SM-SC with $\alpha =3$ and 64-3DCII, the JPGA-ISM using (2, 2, 2) reported in^32^, the SQSM using 32QAM, the QIM-TDC using 128-3DCII. From Fig. 4, it can be seen that, in the BER performances comparisons for various schemes, the SM-EIV-T3DC achieves the significant SNR gain over the SQSM, and the slight better SNR gain over the JPGA-ISM. That is because the SM-EIV-T3DC has the bigger squared MED (i.e., $d_{\min ,{\bar{ \textbf{X}}}}^2 \mathop =2/10$) than the JPGA-ISM (whose squared MED is $d_{\min ,{\bar{ \textbf{X}}}}^2 \mathop =2/10.2426$) and the SQSM (whose squared MED is only $d_{\min ,{\bar{ \textbf{X}}}}^2 \mathop =2/20$).

**Fig. 5:**
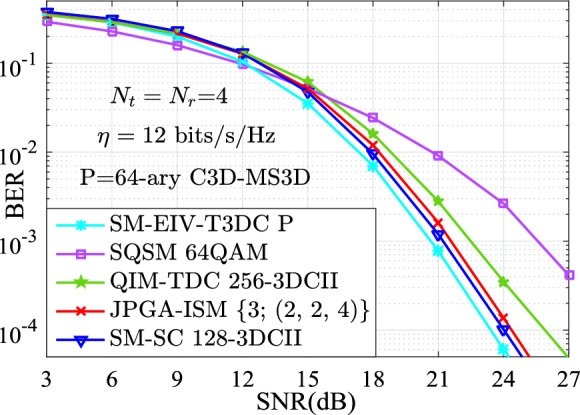
The comparisons of the BER versus SNR performances between the SM-EIV-T3DC and the SM-SC, JPGA-ISM, SQSM and QIM-TDC schemes with $\{N_\textrm{t},N_\textrm{r}\}=(4,4)$ at the SE of 12 bits/s/Hz.

In Fig. 5, the simulation results of the BER versus SNR for various schemes are presented at the SE of 12 bits/s/Hz. According to the Eq. (7), the squared MEDs of the SM-EIV-T3DC using 64-ary C3D-MS3D (64-ary conventional 3D and 64-ary MS3D), the SM-SC with $\alpha =3$ and 128-3DCII, the JPGA-ISM using (2, 2, 4), the QIM-TDC using 256-3DCII, the SQSM using 64QAM are 2/15, 2/16.25, 2/19.0711, 2/38, 2/42, respectively. Obviously, the SM-EIV-T3DC has the advantage of the squared MED. That is to say, the SM-EIV-T3DC achieves the better BER performance. Fig. 5 verifies this result, where about 0.6 dB SNR gain over the SM-SC, 1 dB SNR gain over the JPGA-ISM, about 1.8 dB SNR gain over the QIM-TDC, 5 dB SNR gain over the SQSM are achieved for the SM-EIV-T3DC at the BER value of $10^{-3}$.

**Fig. 6:**
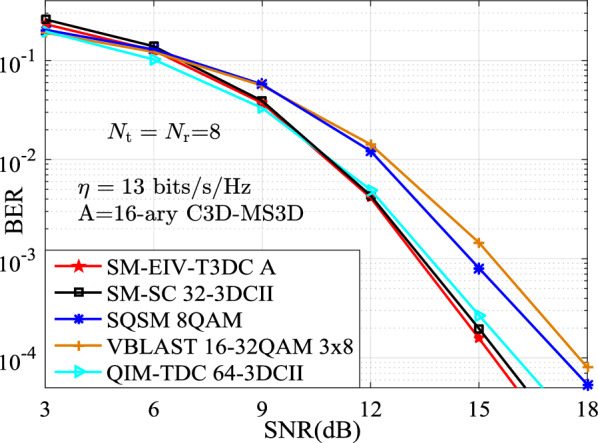
The BER versus SNR performances for the SM-EIV-T3DC, SM-SC, SQSM and QIM-TDC schemes with $\{N_\textrm{t},N_\textrm{r}\}=(8,8)$ at the SE of 13 bits/s/Hz.

**Fig. 7:**
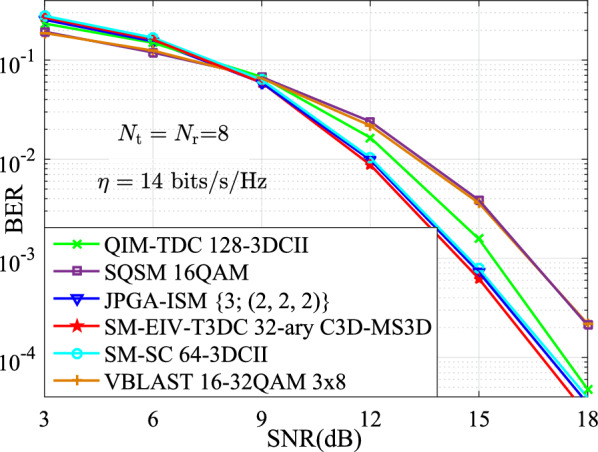
The BER versus SNR performances for the SM-EIV-T3DC, SM-SC, JPGA-ISM, SQSM and QIM-TDC schemes with $\{N_\textrm{t},N_\textrm{r}\}=(8,8)$ at the SE of 14 bits/s/Hz.

**Fig. 8:**
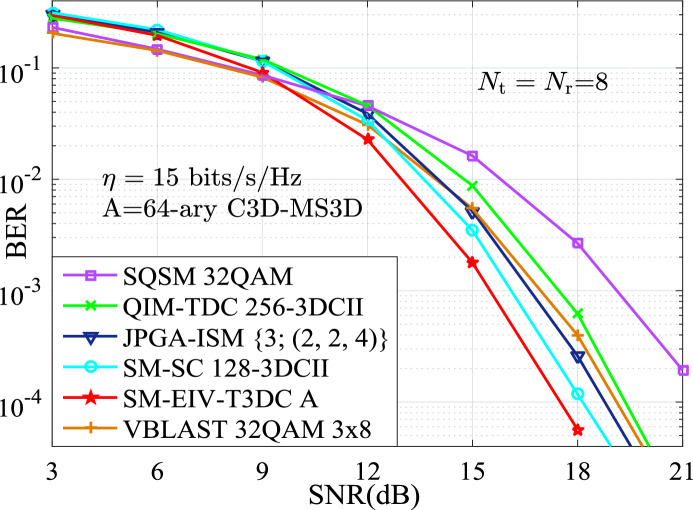
The BER versus SNR performances for the SM-EIV-T3DC, SM-SC, JPGA-ISM, SQSM and QIM-TDC schemes with $\{N_\textrm{t},N_\textrm{r}\}=(8,8)$ at the SE of 15 bits/s/Hz.

Furthermore, Figs. 6, 7 and 8 depict the BER performances of the SM-EIV-T3DC using different modulation orders, i.e., 16-ary C3D-MS3D (i.e., 16-ary conventional 3D and 16-ary MS3D), 32-ary C3D-MS3D and 64-ary C3D-MS3D, and compared with that of various MIMO-IM schemes such as the SQSM, the JPGA-ISM, the SM-SC, the QIM-TDC at the different SEs of {13, 14, 15} bits/s/Hz, the same number of $N_\textrm{t}=N_\textrm{r}=8$ TAs. It can be seen that the better BER performances are achieved for the SM-EIV-T3DC. Specifically, at the SE of 13, 14 bits/s/Hz, it can be observed from both Figs. 6 and 7 that the SM-EIV-T3DC slightly outperforms both of the JPGA-ISM with (2, 2, 2) and the SM-SC with 64-3DCII, but achieves significant SNR gains in comparisons with other schemes such as the SQSM and the QIM-TDC. Similarly, at the SE of 15 bits/s/Hz, the SNR gains achieved by the SM-EIV-T3DC are more significant in comparisons with other schemes. For instance, the SM-EIV-T3DC achieves 5 dB SNR gain than the SQSM at the BER value of $10^{-3}$.

In addition, Figs. 6, 7 and 8 also present that the BER performances of the SM-EIV-T3DC with two or three active TAs are compared with the classic V-BLAST with three TAs. It can be seen that, the SM-EIV-T3DC achieves the significant BER performances in comparison with the classic V-BLAST using {16, 32}QAM.

### Simulation results under imperfect channel state information

To quantitatively assess the robustness, a well-established imperfect CSI model is adopted. Assuming that the estimated channel matrix ${ \hat{ \textbf{H}}}$ at the receiver will be modeled as:

13 $$\begin{aligned} { \hat{ \textbf{H}}} = \textbf{H} + \textbf{E}, \end{aligned}$$

where $\textbf{H}$ is Rayleigh fading channel matrix of Eq. (3). $\textbf{E}$ is the estimation error channel matrix, each entry of which is independent and identically distributed (i.i.d.) complex Gaussian random variables with zero mean and variance $\sigma _e^2$.

Based on the estimated channel matrix ${\hat{ \textbf{H}}}$, the expression of Eq. (4) will be rewritten as

14 $$\begin{aligned} \left[ {\hat{\alpha } ,\hat{\tau } ,\hat{M}} \right] = \arg \mathop {\min }\limits _{\alpha ,\tau ,M} \left\| {\textbf{y} - {\hat{ \textbf{H}}} \cdot \mu \cdot \textbf{X}} \right\| ^2 \end{aligned}$$

**Fig. 9:**
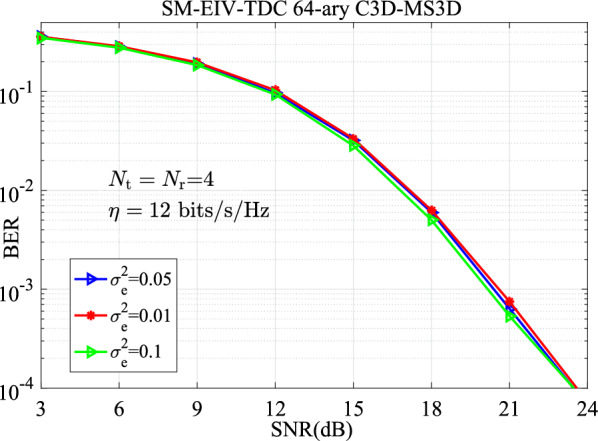
BER curves comparison of the SM-EIV-T3DC over the channel coefficients with different variances of $\sigma _e^2$ at $\{N_\textrm{t},N_\textrm{r}\}\in \{4,4\}$ and 12 bits/s/Hz.

Then, Fig. 9 presents the BER versus SNR curves under multiple levels of channel estimation accuracy (e.g., $\sigma _e^2=0.01,~0.05,~0.1$). These results vividly illustrate the performance degradation of the SM-EIV-T3DC as CSI quality decreases.

**Fig. 10:**
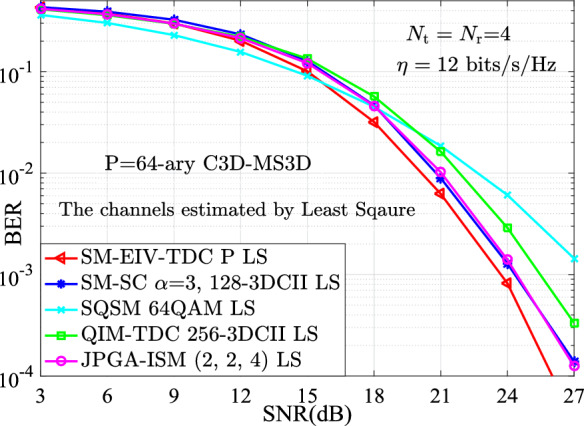
BER curves comparison of the SM-EIV-T3DC over the estimated channel matrix with the LS algorithm at $\{N_\textrm{t},N_\textrm{r}\}\in \{4,4\}$ and 12 bits/s/Hz.

In addition, in the scenario of using the least square (LS) algorithm to estimate the channel coefficients of $\textbf{H}$, the estimated channel matrix can be expressed as

15 $$\begin{aligned} {\hat{ \textbf{H}}}_{\textrm{LS}} \mathrm{= } \textbf{Y}_\textrm{e} \textbf{P}^H \left( {\textbf{PP}^H } \right) ^{\mathrm{- 1}}, \end{aligned}$$

where $\textbf{Y}_\textrm{e} = \textbf{H} \cdot \textbf{P}^H + \textbf{V}$, $\textbf{V} \in C^{N_\textrm{r} \times N_\textrm{t}}$ is an AWGN vector, $\textbf{P} = (1/\sqrt{N_\textrm{t} } ) \cdot \mathrm{hadamard(}N_\textrm{t} \mathrm{)}$ is trained by Hadamard matrix with $N_\textrm{t} \times N_\textrm{t}$ dimensions. On the basis of the estimated ${\hat{ \textbf{H}}}_{\textrm{LS}}$, the proposed SM-EIV-T3DC also has the advantage of the BER performance. For instance, at 12 bits/s/Hz, Fig. 10 presents that the proposed SM-EIV-T3DC with 64-ary C3D-MS3D outperforms at least 0.8 dB over both the SM-SC^30^ with $\alpha =3$ and 128-3DCII and the JPGA-ISM^32^ with (2, 2, 4), about 2 dB over the QIM-TDC with 256-3DCII, at least 3 dB over the SQSM with 64QAM at the BER value of $10^{-3}$.

## Conclusions

In this paper, a new design of the SM-EIV-T3DC, which further exploits more additional information of the spatial domain, is proposed to design the sets of AI vectors with three dimensional framework for enhancing the SE and reliability of wireless transmission. Firstly, the extended set of AI vectors in the quadrature dimension is designed. On the basis of the extended AI vector set, four AI sets are designed with the dimension of 3D constellation. Furthermore, in order to enhance the squared MED, modified secondary 3D constellation is designed. Finally, the BEP is analyzed, the theoretical results are depicted to verify the effectiveness of the SM-EIV-T3DC and simulation results for the SM-EIV-T3DC are depicted to show the advantage of the BER performance in comparisons with other schemes such as the SM-SC, SQSM, JPAG-ISM and QIM-TDC schemes. In addition, to address this practical challenges such as detection complexity and peak-to-average-power ratio (PAPR), our future work will focus on designing the low-complexity near-ML detection algorithms and the development of effective PAPR reduction techniques.

## Data Availability

The datasets used and/or analysed during the current study available from the corresponding author on reasonable request.
